# Intracerebroventricular injection of neuropeptide Y suppresses luteinizing hormone pulses in mice

**DOI:** 10.1111/jne.70265

**Published:** 2026-09-13

**Authors:** Lauren A. Young, Evan R. Hurtado, Kyla D. Jacobs, Zhi Mei Maria Lee, Casey C Nestor, Richard B. McCosh

**Affiliations:** ^1^ Department of Biomedical Sciences, College of Veterinary Medicine and Biomedical Sciences Colorado State University Fort Collins Colorado USA; ^2^ Animal Reproduction and Biotechnology Laboratory Colorado State University Fort Collins Colorado USA

**Keywords:** ICV injection, kisspeptin, luteinizing hormone, neuropeptide Y

## Abstract

Stress impairs reproduction, at least in part, by inhibition of Kisspeptin/Neurokinin B/Dynorphin (KNDy) cells and suppression of gonadotropin secretion. However, the mechanism for this inhibition is not clear. Neuropeptide Y (NPY) is a pleiotropic molecule that is produced in neurons throughout the brain and mediates several physiological processes including energy homeostasis and stress responses. Centrally administered NPY elicits variable effects on luteinizing hormone (LH) secretion, which vary with species, gonadal status, and the site of infusion. Therefore, our objective was to determine the in vivo action of central NPY administration on LH pulses in mice. Frequent blood samples were collected and assayed for LH concentrations from male and female mice that received a central injection of NPY or saline (control). Additional studies were performed in which NPY or saline treated animals received a kisspeptin challenge to evaluate direct effects on GnRH cells, or tissue collection to evaluate transcripts associated with KNDy cells. Intracerebroventricular injection (ICV) of NPY robustly suppressed LH pulses in ovariectomized (OVX) female and gonadectomized (GDX) male mice. However, the same treatment in gonad‐intact males yielded variable responses, and analysis in estradiol‐replaced OVX female mice revealed an unexpected suppression of LH following brief isoflurane exposure. In assessment of KNDy neurons, we found a statistically significant increase in the abundance of mRNA encoding dynorphin in micro‐punches of the arcuate nucleus 1 h after ICV injection of NPY. In further support of the hypothesis that NPY impairs KNDy cells, we found that animals pretreated with NPY or saline had the same response to exogenous kisspeptin, suggesting gonadotropin‐releasing hormone neurons and gonadotrope cells remain fully responsive to stimulation. We conclude that NPY is sufficient to suppress LH secretion in OVX female and GDX male mice and that this suppression is likely mediated by the KNDy cells in the arcuate nucleus.

## 1 INTRODUCTION

Mammals have evolved to prioritize survival in times of adversity. Since reproduction is energetically costly, mechanisms to inhibit fertility during stress (including undernutrition/starvation) have developed. These mechanisms are manifest, at least in part, in the suppression of the hypothalamic–pituitary–gonadal (HPG) axis, often displaying reduced gonadotropin secretion. Indeed, suppression of the pulsatile pattern of luteinizing hormone has been observed in a variety of stress models in many species.^1^ For instance, restraint stress, a type of psychosocial stress, inhibits LH secretion in mice^2^, ^3^ and rats.^4^ Injection of lipopolysaccharide (LPS), an immune/inflammatory stress model, suppresses LH pulses in mice,^5^ rats,^6^ and sheep.^7^ Acute metabolic stress, modeled by rapid decreases in blood glucose concentrations (insulin‐induced hypoglycemia [IIH]) or glucose availability, inhibits the HPG axis and suppresses LH pulses in numerous species, including monkeys,^8^ sheep,^9^ rats,^10^ and mice.^11^ Similarly, undernutrition^12^ and starvation^13^ also impair gonadotropin secretion in mice. Since stress and resource deprivation are pervasive in the modern world, understanding the neuroendocrine mechanisms underlying the suppression of reproduction during adversity is of critical importance.

Neurons in the arcuate nucleus (ARC) that co‐express Kisspeptin, Neurokinin B, and Dynorphin, termed KNDy cells,^14^ form the GnRH pulse generator.^15^, ^16^ These cells are an interconnected population responsible for generating episodic release of GnRH, which in turn stimulates pulsatile luteinizing hormone (LH) secretion.^16^, ^17^ Several lines of evidence support the hypothesis that KNDy neurons become inhibited during stress. First, various stressors have been shown to inhibit the activation of KNDy neurons, as indicated by reduced cFos expression. In OVX mice, fewer ARC kisspeptin cells contained c‐Fos protein following IIH^11^ or LPS.^5^ Similarly, restraint stress decreased the proportion of *Kiss1* cells expressing *cfos* in both OVX female (Yang et al.^2^) and gonadectomized (GDX) male mice (Yang et al.^3^). Second, in addition to reduced neuronal activation, stress also alters kisspeptin transcript expression. The abundance of *kiss1* mRNA in the ARC is significantly decreased in estradiol replaced OVX female rats following IIH.^18^ And finally, the LH response to delivery of exogenous kisspeptin following IIH^11^ or LPS^5^ induced suppression of pulsatile LH secretion remains unimpaired, revealing no deficit in GnRH or gonadotroph cell function. Collectively, these studies highlight the negative effects of stress on KNDy cells; however, the precise mechanisms mediating this inhibition remain a major outstanding question.

Neuropeptide Y is a pleiotropic neuropeptide expressed throughout the brain, including the arcuate nucleus (ARC), medial nucleus of the amygdala, and the nucleus of the solitary tract (NTS).^19^ NPY has been well studied for its role in the regulation of feed intake as a potent orexigenic molecule,^20^ but it is also important for mediating stress responses. There is evidence that NPY contributes to the activation of the hypothalamic–pituitary–adrenal (HPA) axis as central administration of NPY increases corticotropin releasing hormone (CRH) protein in the median eminence,^21^ and infusion of NPY into the paraventricular nucleus (PVN) increases serum corticosterone^22^ in male rats. Additionally, acute restraint stress in rats increases the abundance of mRNA that encodes NPY in several brain regions including the ARC and the medial nucleus of the amygdala.^23^ Taken together, these data demonstrate that NPY can be positively regulated by stress, and is sufficient to activate the hypothalamic–pituitary–adrenal axis.

Several lines of evidence support the possibility that NPY also regulates gonadotropin secretion. Injection of NPY into the 3rd ventricle in OVX sheep significantly increases the LH inter‐pulse interval,^24^ and suppresses LH pulse frequency and amplitude in OVX rats.^25^ In rabbits, the response to infusion of NPY into the median eminence varies with gonad status, in which OVX females displayed reduced LH secretion while ovary intact does had a marked increase in LH secretion.^26^ Furthermore, NPY infused into the stalk median eminence of male and female gonadectomized monkeys stimulated release of GnRH.^27^ These differences may be related to effects of species, sex/sex steroids, and/or sites of NPY injection, and highlight our incomplete understanding of this neuropeptide.

Another approach to investigating the effects of NPY is via chemogenetic or optogenetic activation of cells that produce NPY. To date, neurons in the arcuate nucleus that contain agouti‐related peptide (AgRP) and NPY have received the most attention related to the control of reproduction. These cells coordinate metabolic cues regarding energy status,^28^ and project directly to KNDy cells, thus altering their activity in response to disruptions in energy homeostasis.^29^ Optogenetic activation of ARC AgRP/NPY neurons directly inhibited ARC *Kiss1*‐expressing cells, and chronic chemogenetic activation of these neurons impaired estrus cycles and fertility in female mice.^29^ Additionally, chemogenetic activation of AgRP/NPY neurons slows luteinizing hormone pulsatility in OVX female mice, but not gonadectomized males or gonad‐intact mice of either sex,^30^ reiterating the effect that sex and sex steroids have on reproductive responses.

As a pleiotropic molecule, NPY that is centrally administered elicits variable effects on LH secretion, which are dependent on several factors including species, gonadal status, and the site of infusion. To further understand the role of NPY in the regulation of reproduction in mice, we have conducted a series of studies designed to determine if central injection of NPY suppresses LH pulses, and if that suppression could be mediated by KNDy neurons. First, to test the hypothesis that NPY is sufficient to suppress LH secretion in mice, we evaluated the effects of intracerebroventricular (ICV) injection of NPY on LH secretion in ovariectomized (OVX) females with and without diestrus‐like estradiol replacement, and in gonad intact and gonadectomized (GDX) males. To test the hypothesis that KNDy cells are altered by NPY injection, we assessed the abundance of mRNAs encoding for kisspeptin, neurokinin B and dynorphin. Finally, to test the hypothesis that impairment occurred at KNDy cells rather than in downstream signalling, we determined if GnRH cells were also inhibited by NPY, we evaluated responses to kisspeptin in mice pre‐treated with saline or NPY.

## 2 METHODS

### 2.1 Animals

Adult male and female C57BL/6J mice bred from founders purchased from Jackson Laboratory (stock # 000664) aged 3–7 months were housed 2–5 animals per cage under standard laboratory conditions with a 12:12 light/dark photocycle in our vivarium at Colorado State University. Mice had free access to feed (irradiated laboratory rodent chow; Teklad/Global, 2918) and water at all times including during blood sampling periods. All animal procedures were approved by the Colorado State University Institutional Animal Care and Use Committee and performed in accordance with the National Institute of Health guidelines for the care and use of research animals.

### 2.2 Experimental design

#### 2.2.1 Experiment 1: Does central delivery of NPY suppress LH pulses in female mice?

**FIGURE 1:**
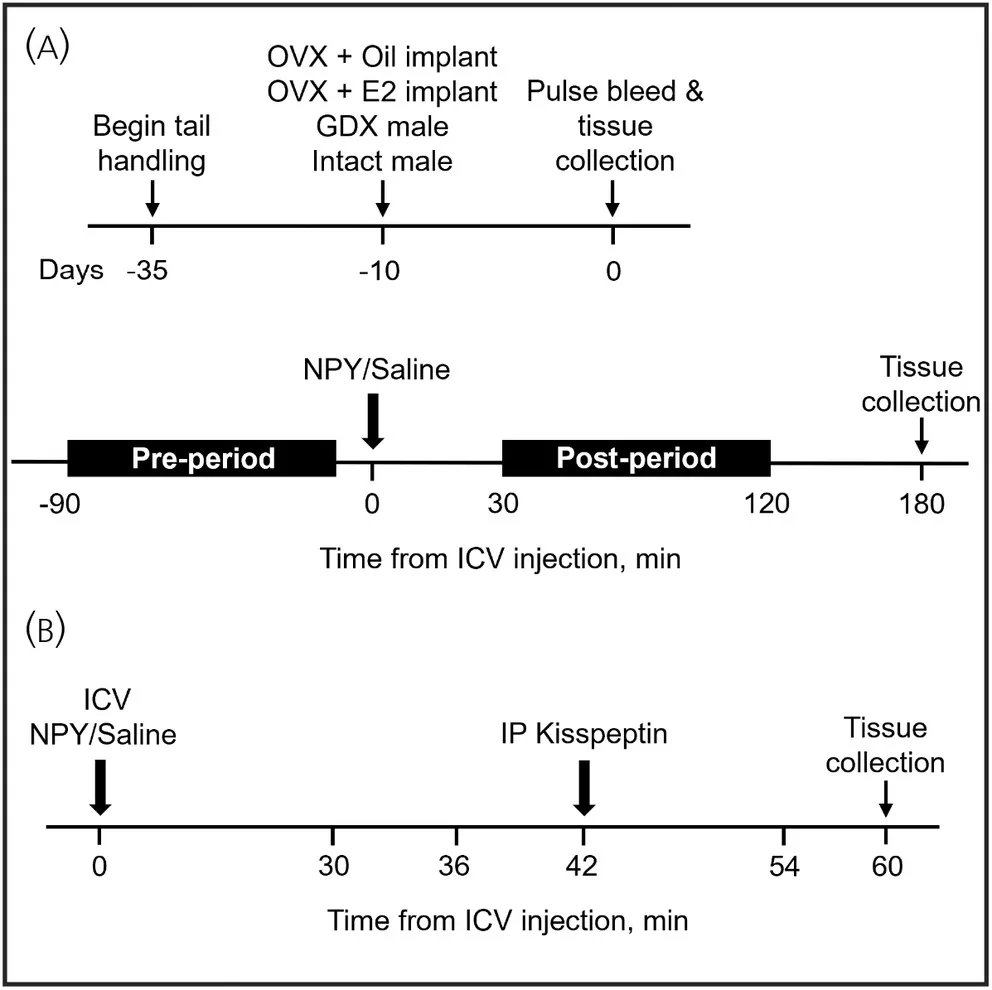
Experimental timeline for Experiment 1 and 3 (A), and Experiment 6 (B). The top panel in A depicts the experimental timeline and the bottom panel depicts the protocol for the pulse bleed and tissue collection on day zero.

To determine if central delivery of NPY suppresses luteinizing hormone pulses in female mice, two endocrine groups were used: (1) OVX with oil (vehicle) implant (OVX + Oil), and (2) OVX with E2 containing oil implant (OVX + E2). Mice were first handled for 5 weeks for acclimatization to tail‐tip blood collection as previously described (McCosh et al.^31^). Ten days prior to blood collection, mice were OVX via bilateral lumbar laparotomy and received a subcutaneous (sq) silastic implant (1.98 mm I.D. [3.18 mm O.D] × 1 cm long) containing either a sesame oil implant (oil) or 100 ng E2 to replace diestrus‐like E2 concentrations. Surgery was performed under isoflurane anesthesia with aseptic technique following administration of analgesic buprenorphine SR (0.012–0.016 mg/kg body weight). The day before blood collection, a craniotomy was performed following buprenorphine SR administration under isoflurane anesthesia with an 18‐gauge needle as previously described.^32^ For the assessment of pulsatile LH secretion, 3 μL blood samples were collected from the tail‐tip at 6 min intervals for 90 min. Mice were then briefly anesthetized with isoflurane and were randomly assigned to receive a free‐hand intracerebroventricular injection^32^ of either NPY (0.5 nmol in 3 μL injection volume) or saline as a vehicle control (3 μL injection volume). For free‐hand injection, fur was clipped from the top of each mouse head, and animals received a craniotomy (18ga needle, 1 mm caudal, and 1 mm lateral to bregma) on the day before blood sampling. On the day of blood sampling, a 5 μL glass syringe with a 27 Ga needle was used to inject the NPY or saline through the craniotomy. Plastic tubing was used to make a depth gauge allowing the needle to extend 3.5 mm. Dose selection was based on previous studies in rats and mice.^33^, ^34^, ^35^, ^36^, ^37^ Mice were allowed 30 min to recover from anesthesia, then frequent blood sample collection was resumed for another 90 min (Figure 1A). Blood samples were immediately placed on ice and stored at −20°C until analysis. Animals were excluded from analysis for insufficient pulses in the pre‐period (OVX + Oil NPY *n* = 1, OVX + E2 SAL *n* = 1, NPY *n* = 2), excessive bleeding after injection (OVX + Oil SAL *n* = 1, NPY *n* = 1, OVX + E SAL *n* = 2), or missed ICV injection (OVX + Oil SAL *n* = 2, NPY *n* = 4, OVX + E2 SAL *n* = 3, NPY *n* = 3). Final analysis included a total of 57 mice (OVX+ Oil: Saline *n* = 10, NPY *n* = 16; OVX + E2: Saline *n* = 17, NPY *n* = 14).

#### 2.2.2 Experiment 2: Does brief anesthesia suppress LH pulses in E2 replaced females?

We identified an unexpected suppression of LH secretion following saline injection in E2‐replaced OVX females (but not in other groups); therefore, we tested the hypothesis that preparation for free‐hand ICV injection alone (i.e. craniotomy and isoflurane anesthesia in the absence of the ICV injection) impairs LH secretion. Mice were prepared exactly as described in Experiment 1 for the OVX + E2 group, except that on the day of blood collection mice did not receive an injection (no needle insertion); instead, they were anesthetized and held in the nose cone in the exact manner necessary for injection. One animal was excluded from analysis due to having insufficient pulses in the pre‐period, resulting in final analysis of 5 mice.

#### 2.2.3 Experiment 3: Does central delivery of NPY suppress LH pulses in male mice?

To determine if central delivery of NPY suppresses LH pulses in male mice, two endocrine groups were used: (1) gonadectomized males and (2) gonad‐intact males. Mice were prepared in the same manner as Experiment 1, except males were gonadectomized (GDX) via a midline incision in the caudal abdomen under isoflurane anesthesia with aseptic technique following buprenorphine injection 10 days before blood collection. Therefore, our objective was to determine the in vivo action of central NPY administration on LH pulses in mice. Animals were excluded from analysis for insufficient pulses in the pre‐period (GDX NPY *n* = 1, intact males SAL *n* = 1, NPY *n* = 1), excessive bleeding after injection (intact male SAL *n* = 1), or missed ICV injection (GDX SAL *n* = 1, NPY *n* = 2, intact male SAL *n* = 2, NPY *n* = 3). Final analysis included a total of 43 mice (GDX: Saline: *n* = 8, NPY: *n* = 6; Intact male: Saline: *n* = 14, NPY: *n* = 15).

#### 2.2.4 Experiment 4: Does central delivery of NPY affect mRNA abundance of KNDy products 3 h after injection?

To determine if central delivery of NPY reduces the abundance of mRNAs that encode the KNDy peptides, the brains of mice used in Experiment 1 and Experiment 3 were collected 3 h after ICV injection. Mice were deeply anesthetized with isoflurane and decapitated, and brains were collected on dry ice and stored at −80°C until analysis. Analysis included 48 mice (6 mice per group, randomly selected from animals included in final LH analysis [experiments 1 & 3]).

#### 2.2.5 Experiment 5: Does central delivery of NPY affect mRNA abundance of KNDy products 1 h after injection?

To determine if central delivery of NPY alters the abundance of mRNA that encode the KNDy peptides 1 h after NPY injection, mice were OVX and received an oil‐containing silastic implant and then received an ICV injection of NPY or saline. One hour after injection, neural tissue was collected and processed as described in Experiment 4. Analysis included all 15 mice allocated to this project (saline: *n* = 7, NPY: *n* = 8).

#### 2.2.6 Experiment 6: Does central delivery of NPY inhibit the LH response to kisspeptin in OVX mice?

To determine whether central delivery of NPY inhibits the LH response to kisspeptin as a proxy for GnRH neuron and gonadotrope function, mice were handled for acclimatization to blood sampling as described in Experiment 1, and to intraperitoneal (IP) injection by briefly scruffing the animal and performing a sham injection with a blunt needle to their abdomen, twice daily. Mice were OVX and received an oil‐containing silastic implant. Ten days later, animals were randomly assigned to receive an ICV injection of NPY or saline; 42 min after ICV injection all mice received an IP injection of kisspeptin (2 μg/g; rat Kisspeptin‐10, Tocris Bioscience, catalog #4243). Blood samples were collected from the tail‐tip at 30, 36, and 42 min after ICV injection (pre‐kisspeptin period; Figure 1B), and again at 54 min after ICV injection (post‐kisspeptin period). Brains were collected as described in Experiment 1 following the last blood sample for determination of injection placement. Analysis included all 14 mice allocated to this project (saline: *n* = 6, NPY *n* = 8).

### 2.3 LH assay for assessment of LH

Biotinylation of Anti‐Human LH 5303 SPRN‐5 was performed using Thermo Scientific EZ‐link NHS‐PEG_4_ Biotinylation kit (catalog # 21455) according to the manufacturer's instructions and as previously described.^38^ Antibody concentration of the biotinylated LH 5303 SPRN‐5 was determined via NanoDrop to be 3.33 mg/mL. The quantification of the ratio of moles of biotin to moles of protein was performed using the Pierce Biotin Quantitation kit (Thermo Scientific, catalog # 28005). The biotinylated antibody was calculated to have 4.7 moles of biotin per mole of antibody.

Measurement of luteinizing hormone was performed via an ultra‐sensitive enzyme linked immunosorbent assay (ELISA) as previously described,^38^ and was immediately analyzed at 490 and 650 nm with a Molecular Devices Versa Max microplate reader. The sensitivity of the assay was 0.1 ng/mL and the intra‐ and inter‐assay coefficients of variation for these assays determined in pooled mouse blood were 5.6% and 23.7%, respectively. Samples were processed in 21 separate assays; all samples from each mouse were assayed on the same plate, and each assay was balanced for treatment group.

### 2.4 Determination of LH pulses

LH pulses were determined using a reformulation of PULSAR^39^ using the parameters in Table 1, and an additional requirement that the amplitude of a pulse had to be greater than the sensitivity of the assay. Assay variability parameters were derived from recently published reports.^40^, ^41^ Peaks that occurred in either the first or last sample within either sampling period were excluded as pulses.

**TABLE 1:** PULSAR parameters.

Variable	OVX/GDX	Intact male
Smoothing fraction	0.7	0.7
Peak split depth	2.5	2.5
Level of detection	0.1	0.1
Amplitude distance	2	2
G1	2.2	6.2
G2	2.7	4.9
G3	1.9	2.5
G4	1.5	1.5
G5	1.2	1.2
Quadratic	0	0
Linear	2.98	2.98
Constant	0.02	0.02

### 2.5 Determination of injection placement and ARC micro‐punch collection

Brains were thawed to −20°C and sectioned (40 μm) with a Leica CM 1950 cryostat until the injection tract was visible. To be considered correctly placed, the injection tract had to intersect the lateral ventricle.

Thick sections (250 μm) were collected throughout the hypothalamus, and those that contained the ARC were selected for microdissection using a clean 2 mm biopsy punch with plunger (Integra, catalog # 33‐31‐P/25) to isolate a semi‐circle of tissue containing bilateral ARC that was stored at −80°C.

### 2.6 RNA extraction, cDNA synthesis, and qPCR

RNA was extracted from ARC tissue using a Zymo Research Quick‐RNA MicroPrep kit (catalog # R1050), which included DNase treatment, per the manufacturer's instructions. cDNA was synthesized using PCR Biosystems UltraScript cDNA Synthesis kit (catalog # PB30.11‐10) per the manufacturer's instructions using 175 ng of RNA.

Quantitative polymerase chain reaction was performed by using PCR Biosystems qPCRBIO SyGreen Blue Mix Lo‐ROX (catalog # PB20.15‐20) reagent with gene‐specific primers (Table 2, ^12^;) and 1 μL of cDNA for each primer set in triplicate on a Roche 480 LightCycler.

**TABLE 2:** Primer sequences for quantitative polymease chain reaction.

Gene	Sequence (5′ → 3′)
*Gapdh*	
Forward	TGCACCACCAACTGCTTAG
Reverse	GGATGCAAGGGATGATGTTC
*Kiss1*	
Forward	CCCTTCCTCCCAGAATGATC
Reverse	TCCCAGGCATTAACGAGTTC
*Tac2*	
Forward	CGTGACATGCACGACTTC
Reverse	CCAACAGGAGGACCTTAC
*Pdyn*	
Forward	AGCAGTAAGCAGGTCATTCATCC
Reverse	CACGCCATTCTGACTCACTTGT

### 2.7 Statistical analysis

For Experiments 1 & 3 the mean LH concentration (mean of every value within the sampling period), mean pulse amplitude (the difference in maximal LH concentration value from that of the preceding nadir), and mean number of pulses per sampling period for each animal was determined. Due to the repeated measures analysis of data with relatively small samples and variable group sizes, we performed a three‐way repeated‐measures permutation ANOVA using the aovperm() function from the permuco R package. Note this approach does not require assumptions related to the distribution of data and is robust to heterogeneity of variance. This analysis was performed separately for males and females; the model included endocrine group (oil vs. E2 implant or GDX vs. testes intact), time (pre vs. post injection) and treatment (NPY vs. Saline). Post hoc pairwise comparisons were performed using the lmperm() function on subsetted data for each combination where appropriate. Animals were excluded from data analysis if they did not have two pulses (females and GDX males) or one pulse (intact males) in the pre‐period, if the injection track missed the lateral ventricle, or if excessive brain bleeding was found at the time of tissue collection. Note, different requirements for the number of pulses in the period based on endocrine group is based on well‐documented differences in secretion patterns among these groups.^42^, ^43^

For Experiment 2, a paired *t*‐test was performed to determine statistical significance from the pre‐period to the post‐period following isoflurane exposure.

For Experiment 4 and 5, qPCR analysis was performed via the delta–delta Ct method using *Gapdh* as the reference gene and compared between treatments (NPY vs. saline) using JMP Statistical Software (Student Edition 19.0.1). Equal variance was assessed by Levene's test and normality was assessed with the Shapiro–Wilk test. Delta–delta CT values were compared between treatments using Student's *t*‐test; a Benjamini–Hochberg correction (with 5% false discovery rate) was used to adjust the critical value for multiple comparisons.

For Experiment 6, the mean of the three pre‐kisspeptin LH values was calculated and compared to the post‐kisspeptin LH value using a two‐way repeated measures permutation ANOVA. Animals were excluded from analysis if the injection they received was a missed injection or if they had excessive brain bleeding.

## 3 RESULTS

### 3.1 Experiment 1: Central NPY administration suppresses LH pulses in female mice

**FIGURE 2:**
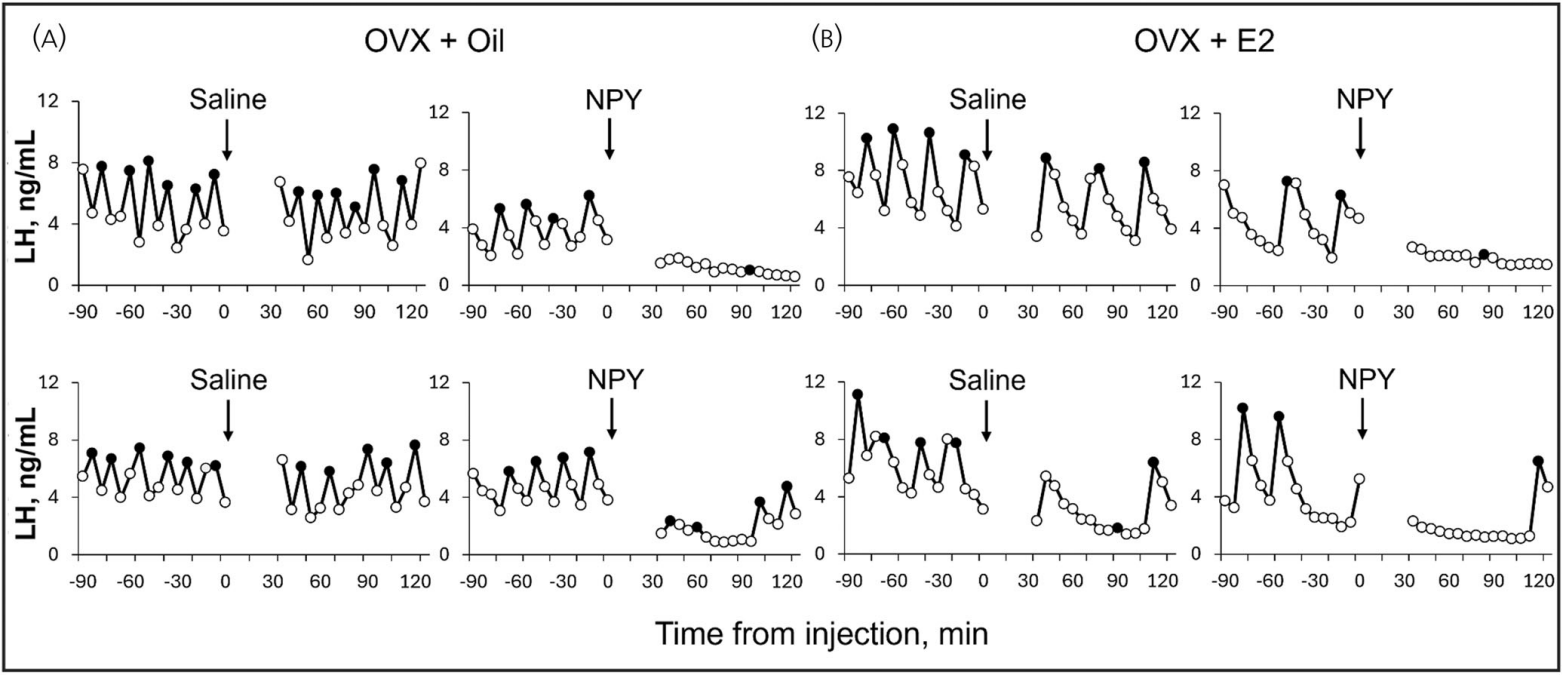
Representative luteinizing hormone pulse profiles in ovariectomized female mice from groups OVX + Oil (A) and OVX + E2 (B). Blood samples were taken prior to (−90–0 min) and following (30–120 min) ICV injection of either NPY or saline. Time of injection with saline or NPY is designated by the arrow (0 min). Filled data points represent pulses.

**FIGURE 3:**
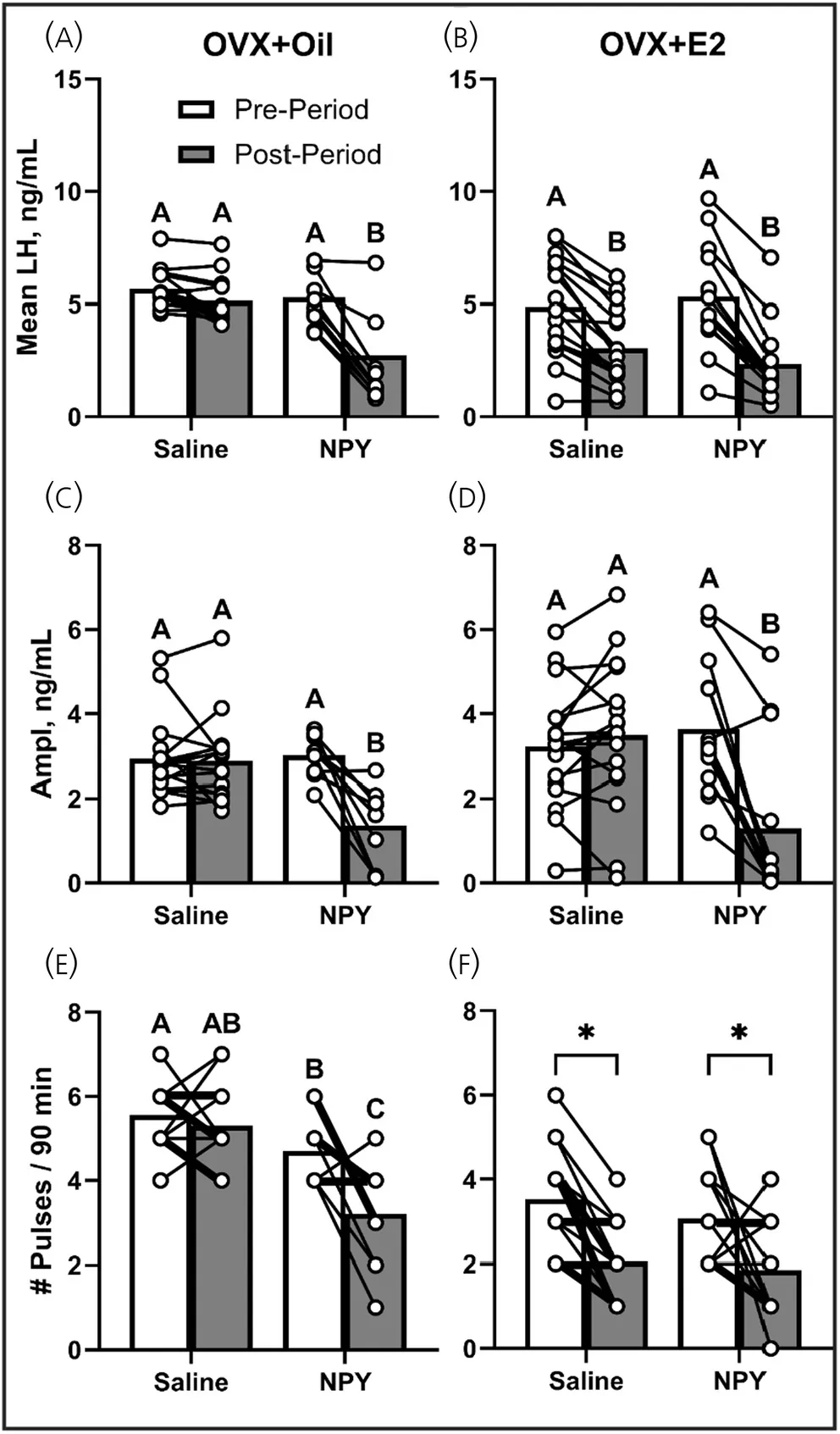
ICV injection of NPY suppresses LH secretion in female mice. Bar plots represent the group means ± SEM for mean LH (A, B), mean pulse amplitude (C, D), and mean number of pulses (E, F). The pre‐injection period is shown in white, and the post‐injection period is shown in grey. Lines connect data points for each animal, heavy line weight indicates multiple animals. Bars with unique letters are statistically significantly different. OVX + Oil, Sal *n* = 16, NPY *n* = 10. OVX + E2, Sal *n* = 17, NPY *n* = 14.

Representative luteinizing hormone pulse profiles for OVX female mice grouped based on steroid status (oil or E2 replaced) are shown in Figure 2. Both groups received a treatment of an intracerebroventricular (ICV) injection of either saline or neuropeptide Y (NPY) as indicated by the arrow (time 0), and LH pulses were evident in all animals in the pre‐period. Group means for mean LH concentration, LH pulse amplitude and number of LH pulses are displayed in Figure 3. In OVX + Oil mice, there was a significant interaction between time and treatment for mean LH (*F*_1,24_ = 22.97, *p* = 0.0001, Figure 3A), pulse amplitude (*F*_1,24_ = 12.23, *p* = 0.0007, Figure 3C), and number of pulses (*F*_1,24_ = 6.15, *p* = 0.0235, Figure 3E), such that these values were significantly reduced following NPY injection but not were not altered after saline injection. In OVX + E2 mice, there was a significant interaction between time and treatment only for mean LH (*F*_1,29_ = 7.65, *p* = 0.0076, Figure 3B) and LH pulse amplitude (*F*_1,29_ = 30.21, *p* = 0.0001, Figure 3D) such that these values were significantly reduced following NPY injection, but were not altered by saline. Unexpectedly, in OVX + E2 mice, mean LH and the number of pulses were decreased after both saline and NPY (i.e., main effect of time: num pulses: *F*_1,29_ = 24.85, *p* = 0.0001, Figure 3F). Interestingly, animals with a suppression of LH pulsatility that were later determined to have an on‐target injection of NPY, experienced a dramatic sickness‐like behavioral response. Approximately 30 min after NPY injection, mice displayed hunched posture, facial grimace, and lethargy that was interrupted by bouts of voracious eating that was starkly different from control mice. Although observations were subjective and taken by an unblinded technician, behavioral responses to NPY were unmistakable in their intensity and consistency.

### 3.2 Experiment 2: Brief exposure to isoflurane anesthesia suppressed LH pulses in E2‐replaced OVX mice

**FIGURE 4:**
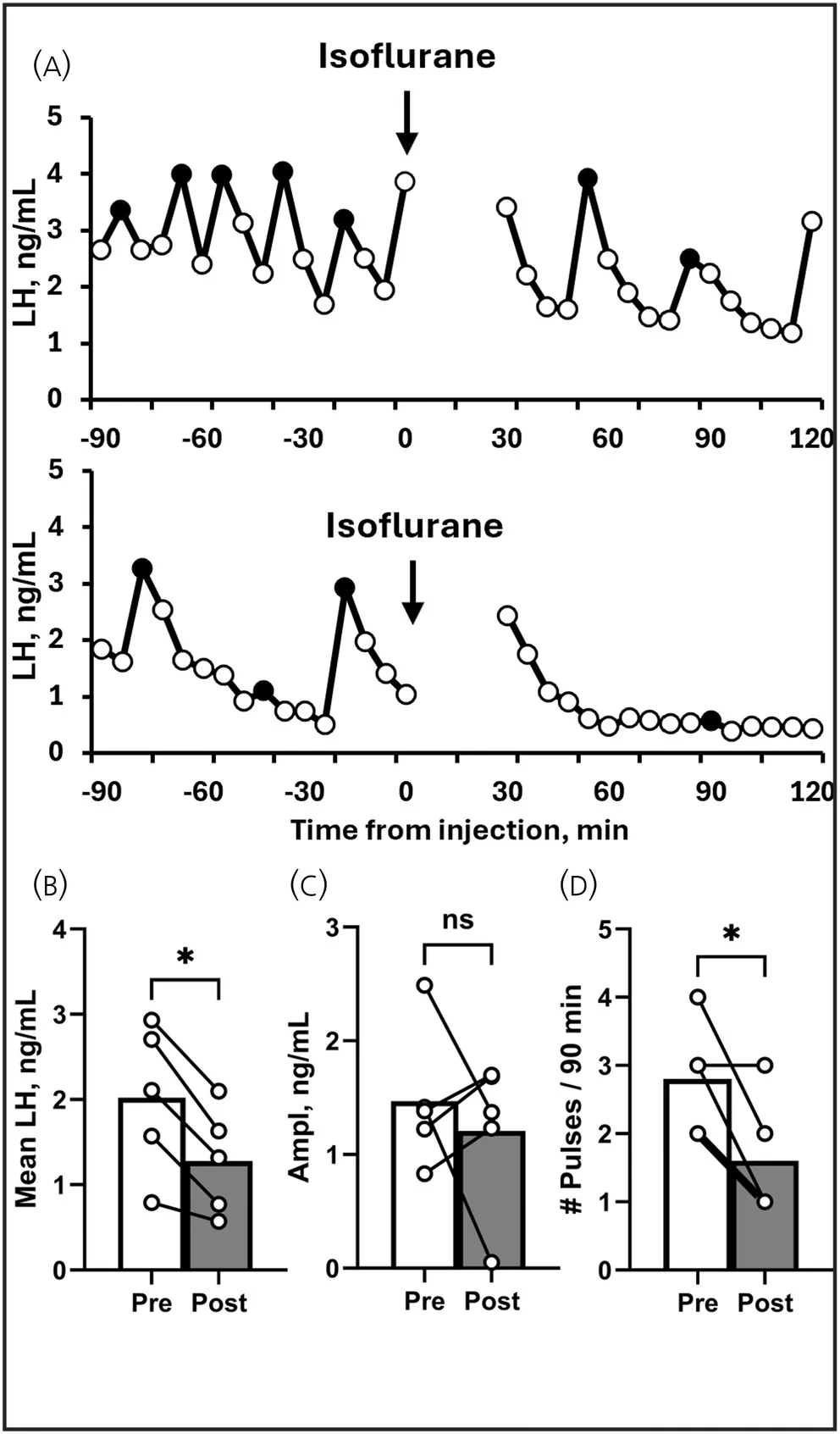
Brief isoflurane exposure suppresses LH secretion in E2 replaced OVX mice. Representative pulse plots from OVX + E2 female mice (A). Time of isoflurane exposure is designated by the arrow. Group means for mean LH (B), pulse amplitude (C), and number of pulses (D). Lines connect data points for each animal; heavy line weight indicates multiple animals. *p ≤ 0.05, *n* = 5.

We observed an unexpected suppression of LH secretion in the saline‐treated OVX + E2 group; thus, we hypothesized that the brief isoflurane exposure necessary to make free‐hand ICV injections is sufficient to suppress LH secretion in the presence of E2. Figure 4A shows representative pulse plots from two mice. LH pulses were clearly evident in the pre‐anesthesia sampling period and were partially or fully suppressed in the majority of animals post isoflurane. Both Mean LH (*p* = 0.0061, Figure 4B) and the number of pulses (*p* = 0.0327, Figure 4D) were significantly lower following anesthesia compared to the pre‐injection period. LH pulse amplitude (Figure 4C) was not altered by isoflurane.

### 3.3 Experiment 3: Central NPY administration suppresses LH pulses in male mice

**FIGURE 5:**
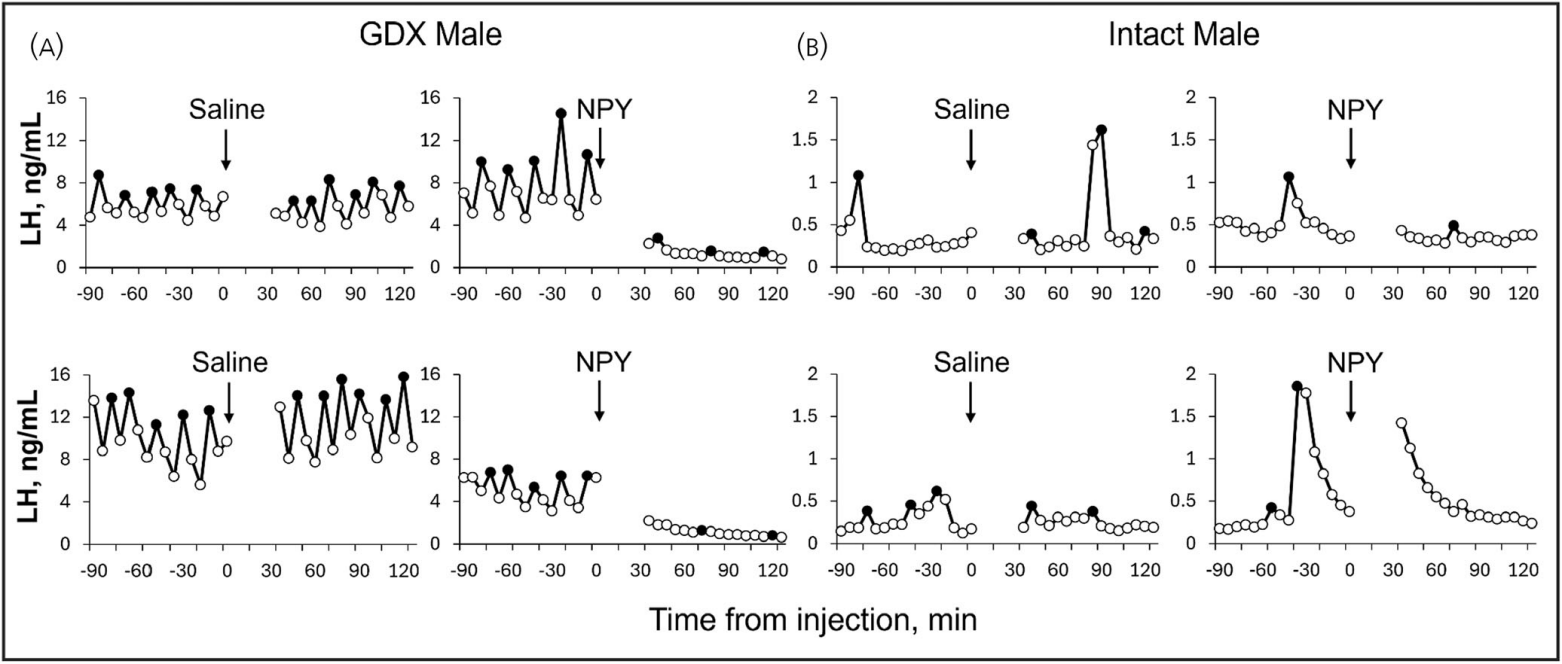
Representative luteinizing hormone pulse profiles in gonadectomized and gonad‐intact male mice. Blood samples were taken prior to (−90–0 min) and following (30–120 min) ICV injection of either NPY or saline. Time of injection with saline or NPY is designated by the arrow (0 min). Filled data points represent pulses.

**FIGURE 6:**
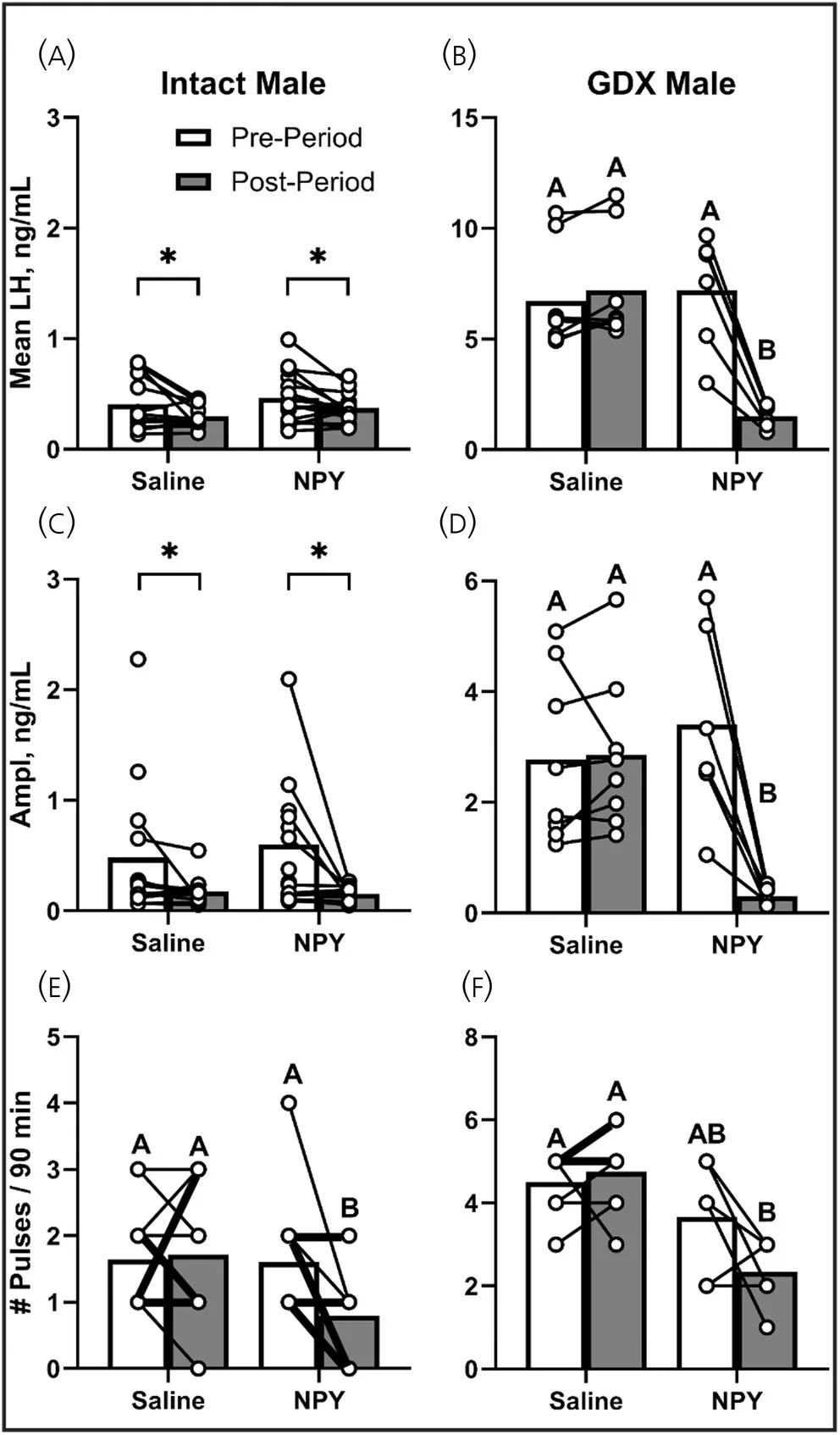
ICV injection of Neuropeptide Y suppresses LH secretion in male mice. Bar plots represent the group Means ± SEM for mean LH (A, B), mean pulse amplitude (C, D), and mean number of pulses (E, F). The pre‐injection period is shown in white, and the post‐injection period is shown in grey. Lines connect data points for each animal, heavy line weight indicate multiple animals. Bars with unique letters are statistically significantly different. GDX Male, Sal *n* = 8, NPY *n* = 6. Intact Male, Sal *n* = 14, NPY *n* = 15.

Representative LH pulse profiles for GDX and testes‐intact male mice are shown in Figure 5. LH pulses are clearly evident in all gonadectomized males in the pre‐period. Post‐NPY injection, LH pulses were robustly suppressed in all GDX males. Mean LH (Figure 6A), pulse amplitude (Figure 6C), and number of pulses (Figure 6E) were significantly reduced following NPY injection, whereas these metrics were not altered following saline injection. In GDX animals, there was a significant time by treatment interaction each for mean LH (*F*_1,12_ = 58.72, *p* = 0.0001), LH pulse amplitude (*F*_1,12_ = 22.86, *p* = 0.0005) and number of pulses (*F*_1,12_ = 4.95, *p* = 0.0431). Intact males displayed inconsistent and infrequent pulses in the pre‐injection period, yet there was a significant time by treatment interaction for mean number of pulses (*F*_1,27_ = 4.74, *p* = 0.036, Figure 6E) such that this variable was reduced following NPY injection, but was not altered by saline. There was no significant time by treatment interaction; however, there was an effect of time for both mean LH (*F*_1,27_ = 7.63, *p* = 0.011, Figure 6B) and mean pulse amplitude (*F*_1,27_ = 6.86, *p* = 0.014, Figure 6D).

### 3.4 Experiment 4: ICV Delivery of NPY does not alter mRNA abundance of KNDy products in mice 3 h after injection

The abundance of mRNA encoding kisspeptin (*Kiss1*), NKB (*Tac2*), or dynorphin (*Pdyn*) was not altered 3 h after injection of NPY compared to saline injection in either OVX + Oil, OVX + E2, GDX males, nor intact males (Table 3).

**TABLE 3:** mRNA abundance of KNDy products 3 h after NPY or saline.

	Relative quantity (Mean ± SEM)	
	Saline	NPY
*Kiss1*		
OVX + Oil	1.15 ± 0.21	1.82 ± 0.37
OVX + E2	1.05 ± 0.18	0.99 ± 0.20
GDX Male	1.08 ± 0.19	0.92 ± 0.13
Intact Male	1.48 ± 0.43	2.20 ± 0.48
*Tac2*		
OVX + Oil	1.10 ± 0.20	1.16 ± 0.17
OVX + E2	1.02 ± 0.09	0.82 ± 0.08
GDX Male	1.03 ± 0.12	1.17 ± 0.06
Intact Male	1.02 ± 0.10	0.95 ± 0.10
*Pdyn*		
OVX + Oil	1.05 ± 0.15	1.08 ± 0.19
OVX + E2	1.06 ± 0.18	1.00 ± 0.11
GDX Male	1.00 ± 0.03	1.31 ± 0.22
Intact Male	1.06 ± 0.16	1.07 ± 0.12

### 3.5 Experiment 5: ICV Delivery of NPY alters mRNA abundance of KNDy products in OVX mice 1 h after injection

**FIGURE 7:**
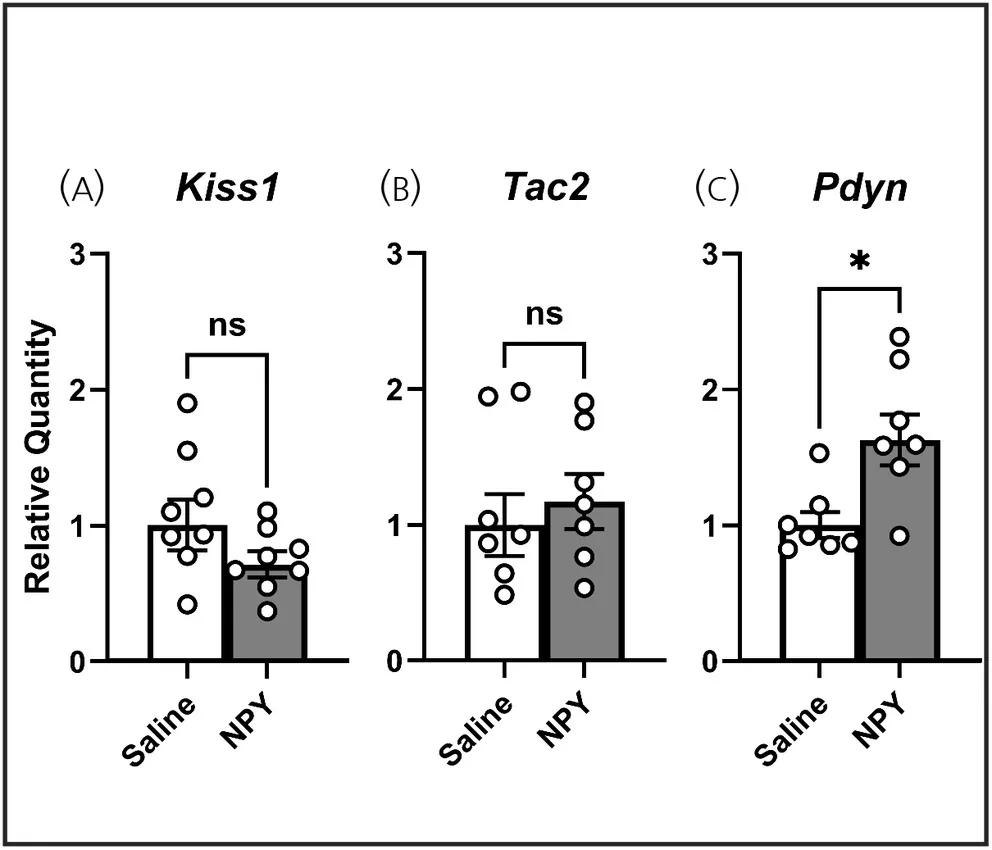
Dynorphin mRNA is increased in NPY‐treated females 1‐h following ICV injection. qPCR analysis of *Kiss1*, *Tac2*, and *Pdyn* abundance in arcuate nucleus 1 h after ICV injection of NPY or saline in OVX + Oil. *p < 0.05. Saline *n* = 7, NPY *n* = 8.

Since LH pulses resumed toward the end of the blood sampling period in several mice (Figures 2 and 5), we reasoned that KNDy gene expression may have recovered by 3 h post injection; therefore, we examined an earlier timepoint (1 h) with consistent suppression of LH pulses. The abundance of mRNA encoding the KNDy peptides is presented in Figure 7A–C. Neither *Kiss1* nor *Tac2* abundance was different between saline and NPY injections. However, *Pdyn* abundance was significantly elevated (1.63‐fold) compared to saline controls (*p* = 0.003, Figure 7).

### 3.6 Experiment 6: ICV Delivery of NPY does not inhibit the LH response to kisspeptin in OVX mice

**FIGURE 8:**
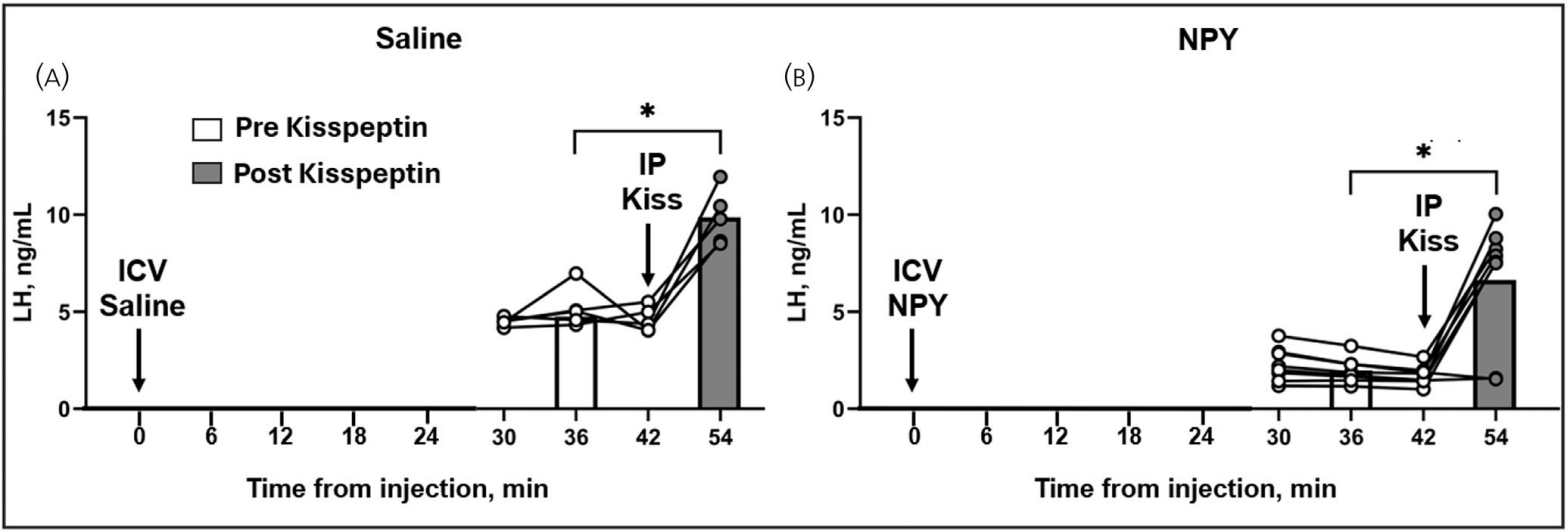
NPY pretreatment does not alter the LH response to kisspeptin in OVX mice. Pre‐treatment with saline is shown in (A). and pre‐treatment with NPY is shown in (B). Pre‐kisspeptin blood samples are shown as open data points. Post‐kisspeptin blood sample is shown as filled (grey) data points. Mean LH from the pre‐injection sampling period (average of three pre‐injection samples, white bars) and from the post‐injection sampling period (grey bars). Saline *n* = 6, NPY *n* = 8.

The experimental timeline is presented in Figure 1B. Mean LH concentrations before and after kisspeptin injection in mice pre‐treated with saline or NPY are shown in Figure 8. By two‐way permutation‐based ANOVA, there was not a statistically significant interaction between time (before vs. after kisspeptin) × pre‐treatment (saline vs. NPY; *F*_1,11_ = 0.08, *p* = 0.7786), but there was a significant main effect of time, indicating that the LH response to exogenous kisspeptin was not different between animals pre‐treated with NPY or saline. Pre‐kisspeptin LH levels were numerically lower in NPY treated animals compared to saline pretreated animals, confirming effectiveness of NPY to suppress LH secretion seen in Figure 3.

## 4 DISCUSSION

In the present study, we aimed to test the hypothesis that central administration of neuropeptide Y suppresses LH pulsatility in female and male mice, and that this effect is mediated via the KNDy cells in the ARC. We first demonstrated that ICV injection of NPY in OVX mice caused robust suppression of LH secretion. Further inquiry revealed that E2 replaced OVX mice are sensitive to the handling and isoflurane anesthesia necessary to make ICV injections, which complicated analysis of NPY effects in these animals. Mice that were GDX also displayed robust suppression of LH secretion, while gonad‐intact males had variable responses to NPY injection that include decreased mean LH and pulse amplitude in response to isoflurane. The abundance of the mRNA that encodes the inhibitory peptide, dynorphin, is significantly increased in the ARC of OVX mice 1 h following delivery of NPY, providing evidence of molecular changes in the ARC coincident with the suppression of pulsatile LH secretion. In further support of the hypothesis that KNDy cells are the primary site of impairment within the HPG axis, we demonstrated that the LH response to exogenous kisspeptin is not altered by NPY injection, suggesting that the NPY‐induced suppression of LH is mediated upstream of KISS1R activation. Taken together, these data indicate that ICV delivery of NPY suppresses LH secretion via central mechanisms in both GDX male and OVX female mice.

Previous work has revealed varying effects of NPY on LH secretion in different species. In our study, we observed robust suppression of LH secretion following ICV injection of NPY in OVX mice, which is similar to reports in some species. Notably, central delivery of NPY also suppresses pulsatile LH secretion in OVX sheep,^24^ rats,^25^ and rabbits.^26^ In contrast, NPY stimulates LH secretion in OVX monkeys^27^ and ovary‐intact rabbits.^26^ Whether this response is purely different among species or is caused by the site of action (NPY was infused into the stalk median eminence in monkeys and rabbits) remains an outstanding question. We also observed a robust suppression of LH pulses in GDX male mice. The only other investigation of central NPY on LH secretion employing GDX males was performed in monkeys, in which stimulatory effects of NPY were noted.^27^

There were distinct differences in the effects of LH suppression based on steroidal status in both male and female mice in our study. In gonad‐intact males, we observed a suppression in the number of LH pulses following NPY that was not observed following saline treatment. In contrast, a reduction in mean LH and pulse amplitude was observed following both saline and NPY (i.e., main effect of time), which precludes interpretation of effects of LH secretion on these metrics. A subset of intact males had LH pulse amplitudes near the sensitivity of the assay (0.1 ng/mL) in the pre‐injection period; whether these reflect real pulses or a random fluctuation that crossed our threshold of pulse detection is not known. An additional challenge with interpretation of these data is that baseline LH concentrations in intact males are low, which makes detecting marked reductions in LH secretion impossible due to a potential floor effect in LH secretion. We note that the parameters for PULSAR analysis that we used were validated in an LH assay that had a greater sensitivity than our assay, and thus may lead us to not detect some low amplitude pulses.

Interpretation of the effects of NPY in E2‐replaced OVX mice was complicated by an unexpected suppression of mean LH and number of LH pulses in the saline‐treated animals. This result led us to test the effects of handling and isoflurane exposure without any ICV injection, which was found to be sufficient to suppress mean LH and the number of pulses. Thus, the reduction in mean LH and number of pulses observed following ICV injection was likely caused by handling and isoflurane exposure, precluding analysis of modulation of these features by E2. However, handling and isoflurane alone did not alter LH pulse amplitude, which was found to be decreased following injection of NPY but not saline. So, we can infer that NPY suppresses LH pulse amplitude in OVX + E2 animals in a similar manner to OVX mice.

The unexpected suppression of LH secretion in E2‐replaced OVX mice exposed to handling and isoflurane could reflect increased sensitivity to mild stress associated with handling or an interaction with isoflurane. An increased sensitivity to handling stress caused by E2 was first noted by the Knobil Laboratory, as they were able to detect LH pulses and multi‐unit activity (MUA) associated with the GnRH pulse generator in OVX monkeys restrained in primate chairs, but not in ovary‐intact animals.^44^ However, LH pulses and MUA activity were detectable in ovary‐intact monkeys that were unrestrained. There are several other examples demonstrating that stress or stress‐associated molecules and pathways impair LH secretion only in the presence of E2. For example, chronic corticosterone treatment suppressed mean LH and LH pulse frequency in OVX + E2 but not OVX mice,^42^ and peripheral delivery of IL‐1β suppressed LH secretion in OVX + E2 but not in OVX mice.^45^ Recently, the Herbison Laboratory demonstrated that chemogenetic activation of ARC projecting catecholamine neurons in the locus coeruleus suppresses synchronized events in ARC kisspeptin cells in ovary‐intact, but not OVX mice.^46^ However, the handling required for ICV injections in the present study is quite brief (removal from cage and placement in an induction box), which is not dissimilar to the handling required for blood sampling, which the mice are well accustomed to before the experiment.

The possibility that anesthetics can alter gonadotropin secretion has been examined in a number of studies. Pentobarbital is well known for its ability to suppress LH pulses in several species.^47^, ^48^, ^49^ Inhaled anesthetics, however, have more varied effects. In women, halothane did not alter LH secretion during surgery (30–60 min) or after surgery (5 and 48 h).^47^ However, in testes‐intact male rats chronic (1 h/day, 25 days) isoflurane exposure reduced GnRH content in the median eminence and reduced *Kiss1* mRNA abundance in the ARC.^50^ Further, in OVX mice, LH pulses are blocked by 30 min of isoflurane anesthesia.^51^ In our experiment, mice were anesthetized for ~4 min to deliver ICV injections and were allowed 30 min to recover from anesthesia before blood sampling resumed. We have not examined the extent and/or duration of LH pulse disruption by our brief isoflurane exposure in OVX mice, but pulses clearly resume in the saline treated animals by the time we collected samples 30 min following injection (Figure 2). Although the mechanism for suppression of LH secretion during isoflurane and the influence of E2 remains an outstanding question, it highlights an important methodological limitation. Thus, there is a technical need to refine procedures for central drug delivery to permit evaluation of gonadotropin secretion in steroid‐treated/ovary intact mice.

Administration of agents intracerebroventricularly enables an efficient way to test widespread effects throughout the brain as the ventricular system flows unidirectionally, from the lateral ventricles toward the spinal canal. Given the clear effects of NPY on LH secretion we observed, a next logical question is where does NPY act? Based on previously published data the primary candidates are the POA, ARC, and PVN. Amphipathic neuropeptides like NPY injected into the cerebrospinal fluid will likely act in regions within 2 mm of the ventricular system,^52^ which includes these candidate regions. The first possibility is the GnRH neurons, as their somas reside in the preoptic area and their fibers project to the median eminence, both regions located within 2 mm of the 3rd ventricle. In mice, GnRH neurons are directly sensitive to NPY with effects varying with receptor subtype (Y1R inhibitory and Y4R stimulatory).^53^ So, while it is possible that ICV administration of NPY could act directly on GnRH neurons, our kisspeptin challenge data support the hypothesis that NPY acts upstream of KISS1R activation, thus the PVN and/or ARC are more likely candidates.

Another plausible site for ICV NPY action is the PVN, which contains a diverse set of neuroendocrine neurons that could be influenced by NPY signaling. Previous immunohistochemical studies in rats^54^, ^55^, ^56^ and mice^57^, ^58^ have demonstrated expression of the NPY receptor subtypes Y1R and Y5R within the PVN. Further, direct infusion of Y1R/Y5R agonists into this region in rats abundantly increases cFos labeling in PVN neurons,^54^ including CRH‐positive cells, and elevates plasma corticosterone, similar to effects seen with ICV NPY administration.^59^, ^60^ It is possible that activation of the HPA axis could contribute to the suppressive effects on LH secretion as elevated corticosterone levels can inhibit the HPG axis. However, corticosterone suppresses LH in OVX + E2 but not in OVX mice,^42^ whereas our data demonstrate clear effects of NPY in the absence of E2. Therefore, glucocorticoids, themselves, are unlikely to mediate the suppressive effects of NPY on LH secretion.

NPY may act in the ARC, which contains several populations of neurons that express NPY receptors. First, the KNDy cells express mRNA transcripts encoding *Npy1r*, *Npy4r*, and *Npy5r* in OVX mice.^61^ Consistent with this, Hessler et al.^62^ demonstrated that the NPY‐induced inhibition of KNDy neuron firing in brain slices was partially blocked by a NPY1R antagonist. These data indicate that KNDy cells express functional NPY receptors. Second, the network of neurons involved in the regulation of feed intake, including AgRP/NPY neurons in the ventromedial ARC and pro‐opiomelanocortin (POMC) neurons in the ventrolateral region both express *Npy1r* and *Npy2r*, and have close contacts with cells in these regions also expressing these receptors.^57^, ^63^, ^64^, ^65^, ^66^ Further, AgRP/NPY neurons send direct projections to KNDy cells to inhibit their activity.^29^ Collectively, these findings suggest that NPY could modulate KNDy neuron activity through both direct and indirect mechanisms within the ARC.

Regardless of where NPY acts (i.e., in the POA, PVN, or ARC), ultimately NPY induces cellular changes in the ARC. Our qPCR data showed a statistically significant increase in the abundance of *Pdyn* mRNA 1 h after NPY delivery. Since dynorphin is an inhibitory peptide with a traditional role in pulse termination^17^ and establishing inhibitory tone at the onset of a pulse of kisspeptin,^67^ it is plausible that NPY induces an upregulation of dynorphin expression to suppress pulse generation and/or the amplitude of pulses that are generated. No changes in *Kiss1 or Tac2* mRNA abundance were observed; however, altered secretion patterns of these peptides is likely given their role in regulating LH pulses.^17^ No differences in mRNA levels for any of the KNDy peptides were observed 3 h following NPY injection, possibly because animals resumed LH pulsatile secretion by that timepoint (Figure 2A, lower right panel shows an animal with LH pulses that resumed by 120 min following NPY). In contrast, NPY injected into the 3rd ventricle of testes‐intact male rats significantly increased *Kiss1* and *Tac2* mRNA levels and reduced *Pdyn* mRNA.^68^ This discrepancy may reflect differences in sex, steroid hormone status, or species, as well as the specific timepoints we assessed.

Following NPY injection, we noticed increased feeding consistent with the well‐established role for NPY secreted from NPY/AgRP neurons in the ARC inducing feed intake.^20^, ^69^ Although feed intake was not quantified in this study, it was an obvious behavior that was observed while collecting blood samples. Although a link between metabolism and reproduction is well established, we think the suppression of LH observed here is due to actions of NPY on neural systems regulating GnRH release, rather than increased feed intake per se. Moreover, we would speculate that performing this experiment in fasted mice may augment the LH response as feed deprivation during increased hunger/motivation to eat induced by NPY may engage psychogenic stress neuropathways, which could be tested in future work. Fasting mice for 5 h during the lights‐on period in mice (the inactive phase when little eating is expected) is sufficient to modestly suppress LH pulses.^11^ Therefore, the present study was conducted in mice with free access to feed in an attempt to minimize potentially confounding or complicating reactions.

We also noted a distinct sickness‐like behavioral phenotype in mice following NPY administration including hunched posture, facial grimace, and lethargy. In review of notes taken by a non‐blinded experimenter, it was found that these sickness‐like behaviors were tightly correlated with the magnitude of suppression of LH pulses. When sickness‐like behavior ceased before the end of the sampling period, it was later found that pulses also resumed at approximately that time. Although these observations were subjective, we could reliably predict whether an injection was correctly placed based on the behavioral response of the mouse. Notably, treatment with LPS,^70^ insulin (insulin‐induced hypoglycemia, unpublished), and chemogenetic activation of catecholamine neurons in the nucleus of the solitary tract^40^ induce similar sickness‐like behaviors in mice. We are not aware of any other description of this sickness‐like phenotype in the literature. However, a variety of behavioral phenotypes have been described following chemogenetic or optogenetic activation of the AgRP/NPY neurons in the hypothalamus, including increased locomotor activity in mice in the absence of food,^20^ suppressed innate fear and anxiety responses^71^ and enhanced wakefulness and decreased duration and integrity of sleep.^72^ Thus, the range of functions that NPY and/or the cells that secrete NPY mediate is quite large.

In summary, these data demonstrate that an ICV injection of NPY is sufficient to suppress pulsatile LH secretion in mice in the sex steroid‐free condition. Moreover, this impairment in LH secretion is likely mediated by the KNDy cells in the ARC, as evidenced by changes in the abundance of mRNA encoding the inhibitory peptide dynorphin, as well as the unaltered LH response to exogenous kisspeptin following NPY‐induced suppression of LH. Together, these data provide more evidence for the functional link between NPY and the regulation of pulsatile gonadotropin secretion.

## AUTHOR CONTRIBUTIONS

**Lauren A. Young:** Investigation; writing – original draft; conceptualization; writing – review and editing. **Evan R. Hurtado:** Writing – review and editing; investigation. **Kyla D. Jacobs:** Investigation; writing – review and editing. **Zhi Mei Maria Lee:** Investigation; writing – review and editing. **Casey C Nestor:** Conceptualization; writing – review and editing. **Richard B. McCosh:** Conceptualization; investigation; writing – original draft; funding acquisition; supervision; writing – review and editing.

## CONFLICT OF INTEREST STATEMENT

The authors declare no conflict of interest.

## Data Availability

The data that support the findings of this study are openly available in Dryad at https://doi.org/10.5061/dryad.bcc2fqzt4.
